# Energy-Balancing Unequal Clustering Approach to Reduce the Blind Spot Problem in Wireless Sensor Networks (WSNs)

**DOI:** 10.3390/s18124258

**Published:** 2018-12-04

**Authors:** Nazmul Islam, Saurabh Dey, Srinivas Sampalli

**Affiliations:** Faculty of Computer Science, Dalhousie University, 6050 University Ave., PO Box 15000, Halifax, NS B3H 4R2, Canada; Saurabh.Dey@dal.ca

**Keywords:** WSN, unequal clusters, blind spot, Sierpinski triangle, cognitive partitioning, energy balancing

## Abstract

Wireless Sensor Networks (WSNs) have become a significant part of surveillance techniques. With unequal clustering approaches and multi-hop communication, WSNs can balance energy among the clusters and serve a wide monitoring area. Recent research has shown significant improvements in unequal clustering approaches by forming clusters prior to the selection of cluster heads. These improvements adopt different geometric fractals, such as the Sierpinski triangle, to divide the monitoring area into multiple clusters. However, performance of such approaches can be improved further by cognitive partitioning of the monitoring area instead of adopting random fractals. This paper proposes a novel clustering approach that partitions the monitoring area in a cognitive way for balancing the energy consumption. In addition, the proposed approach adopts a two-layered scrutinization process for the selection of cluster heads that ensures minimum energy consumption from the network. Furthermore, it reduces the blind spot problem that escalates once the nodes start dying. The proposed approach has been tested in terms of number of alive nodes per round, energy consumption of nodes and clusters, and distribution of alive nodes in the network. Results show a significant improvement in balancing the energy consumption among clusters and a reduction in the blind spot problem.

## 1. Introduction

With the advancement of communication and sensor technologies, it has become possible to develop low-cost circuitry to sense and transmit the state of surroundings. Wireless networks of such circuitry, namely Wireless Sensor Networks (WSNs), can be deployed in a wide variety of applications such as healthcare [1], smart industries [2,3], environmental sensing [4], smart farming [5], and military defense [6]. In addition, WSNs play a significant role in emerging technologies, e.g., big data [7], cloud [8], and the Internet of Things (IoT) [9]. However, they have unique design challenges due to their severe computation and energy constraints [10,11]. Consequently, energy-efficient cluster-based routing [12] and security [13] are still receiving enormous interest from the researchers today.

Typically, a WSN consists of hundreds or thousands of sensor nodes and Base Stations (BSs). Nodes in WSNs can be homogeneous or heterogeneous [14] and communicate with the BS individually or may form several clusters with Cluster Heads (CHs). Communication among the nodes and the BS can be established by a single or multi-hop path link. The limitation of single-hop communication is that nodes far from the BS lose energy rapidly due to the long communication range. On the other hand, nodes closer to the BS die quickly in multi-hop communication since they forward all packets of the network to the BS. This scenario is known as the hotspot problem and several approaches have been proposed to mitigate it by making the clusters unequal in terms of size. Among the unequal clustering approaches, fractal-based ones [15,16] have shown significant improvement in performance as they form clusters prior to the selection of CHs. Although these approaches successfully manage the hotspot problem, they suffer from the blind spot problem. This problem refers to the inability to capture events due to the presence of dead nodes in the network. The lifetime of a network can be divided into two states: (1) the steady state, when all nodes are alive; and (2) the declining state, when nodes start dying. As nodes are uniformly distributed, the network achieves high performance by capturing the desired number of events per unit of time in the steady state. In the declining state, the network cannot capture events uniformly as there persist dead nodes in the region. Hence, the performance of the network continuously degrades once the nodes start dying. For maintaining the performance, it is important to minimize the blind spot problem by shortening the declining state, which is lacking in a majority of existing works.

This paper proposes a cognitive partition-based unequal clustering approach to address the blind spot problem in WSNs. In addition to smaller closer clusters, the proposed approach ensures size-based balanced energy consumption. The selection process of CHs in the proposed approach is divided into two layers, such as weight-based selection of candidate CHs and cumulative distance-based CH selection for each cluster from the candidates. The proposed approach guarantees CHs to have short distances among them and consumes the least energy for packet forwarding. As a result, the lifetime of the network increases with a longer steady state than the declining state that reduces the blind spot problem in the network. The contribution of this paper is novel in the following aspects-

- Partitioning the network in a cognitive way to specify the size of the clusters for balanced energy consumption.
- Adoption of two-layered scrutinization for the selection of CHs to guarantee minimal energy loss from the network.
- Shortening the duration of declining state to reduce the blind spot problem.

The rest of the paper is organized as follows. Section 2 reviews the recent research on unequal clustering approaches. Section 3 describes the proposed approach in detail. Simulation results are given in Section 4 and conclusions are drawn in Section 5.

## 2. Literature Review

Previous works on unequal cluster formation in WSNs can be broadly categorized into probabilistic, deterministic, and preset approaches. CHs are determined randomly in probabilistic approaches, whereas deterministic approaches adopt weight functions, fuzzy logic, heuristic techniques or a hybrid of these to determine the same. The preset approach simplifies its process by predetermining node locations, clusters, and CHs. The proposed approach presented in this paper would be a new addition to the hybrid unequal clustering approaches as it includes both optimization and weight-based approach to select CHs. Clustering in WSNs is typically done for preserving network lifetime by balancing the energy consumption. However, there are other approaches besides clustering that achieve the same goal. For example, the approach presented in Ref. [17] uses cooperative multiple input multiple output (MIMO) technique to minimize energy consumption. It compares the proposed technique with both single-hop and multi-hop communications and proves the efficiency of the proposed approach. However, the recent works on deterministic unequal clustering approach are described below only as they closely relate to the proposed approach.

Several clustering approaches [18,19,20,21,22,23,24,25,26] have been proposed recently based on the weight-based technique. Among them, the proposed approach presented in Ref. [18] divides the monitoring area into fixed sized grids and selects the CHs based on the distance to the midpoints of the grids. Similarly, the proposal in Ref. [19] divides the monitoring area into fixed sized zones and selects the CHs based on the residual energy. Again, the approach presented in Ref. [20] partitions the monitoring area into several hierarchical levels. It adopts a mathematical approach to construct unequal sized clusters; thus, improving the network lifetime. The proposal in Ref. [21] selects CHs through two steps, such as the random selection of tentative CHs and the selection of final CHs. Here, the tentative CHs are selected based on a probability model and the final CHs are selected based on their residual energy. In this approach, each sensor node preserves the minimum number of hop count to the BS that gives the optimal radius of a cluster. The approach presented in Ref. [22] selects its CHs based on the residual energy and distance to the BS. However, it only triggers the selection process once the residual energy of any current CH falls below a threshold level. It also employs relay nodes for CHs having a distance to the BS higher than a predefined value. The proposal in Ref. [23] determines cluster sizes based on the distance to the BS. It uses Dijkstra’s algorithm to find the shortest path route to the BS. The approach presented in Ref. [24] spatially distributes the clusters to balance the energy consumption in the network. For this purpose, it creates tracks around the BS where same sized clusters are formed in the same track. This approach considers the residual energy to select the candidate CHs. Final CHs are selected later from the candidates based on a distance metric rule. The proposed approach in Ref. [25] selects CHs based on the residual energy and coverage area, i.e., the more a node’s sensing area covered by its neighbors, the higher its probability to be a CH. Finally, the approach in Ref. [26] considers the average energy of neighbor nodes beside of a node’s residual energy to select it as a CH. Cluster formation in all these approaches are similar to LEACH [27] and actuated after the selection of CHs. Moreover, Ref. [28] proposes an energy-efficient multi-level and distance-aware clustering mechanism for WSNs which is also a recent advancement in this stream. This approach divides the monitoring area into three logical layers based on the hop count from the base station.

Fuzzy logic is also used in a lot of protocols [29,30,31,32] for making decisions effectively, i.e., selecting the CHs and determining cluster sizes. For this purpose, it takes input parameters such as distance to the BS, centrality, distance from the neighbors, node degree, residual energy, etc., and outputs CH selection probability and cluster size. The approach proposed in Ref. [29] uses fuzzy logic for selecting the CHs and determining radii of the clusters. Here, the input parameters are the distance to the BS, node density, and residual energy, whereas the outputs are clusters’ radii and the probable CHs. The final CHs are determined by a competition that requires an exchange of messages. This approach uses Ant Colony Optimization (ACO) [33] to find the shortest path from a CH to the BS. The proposed approach in Ref. [30] uses Fuzzy Inference System (FIS) [34] to select the CHs in a distributed way that takes residual energy, link quality, and centrality of the node as inputs. This approach has made a significant improvement in WSN reliability by considering link quality while selecting the CHs. The fuzzy output is a value that indicates the probability of a node to become a CH. This approach also uses the scatter factor and the distance of a hypothetical hexagon to the BS for determining the number of CHs in that hexagon. The scatter factor is defined as the average distance of each node to its neighbor nodes in the hexagon. The higher the scatter factor, the more the CHs are required in that hexagon. The proposal in Ref. [31] uses a probabilistic method to determine the tentative CHs and fuzzy logic to finalize the competition radii by considering node degree, residual energy, and distance to the BS. Node degree and residual energy are used again to determine the final CHs. In this approach, nodes can join a cluster based on CH’s degree and distance to the BS. Finally, Ref. [32] presents an approach that takes the same input as FIS to determine both the CHs and cluster sizes. Clustering approaches associated with all these techniques are also similar to LEACH [27], i.e., clusters are formed after the selection of CHs.

Recently, several proposals have been made based on heuristic unequal clustering approach [16,35,36,37,38,39]. The approach presented in Ref. [35] computes the number of CHs and their positions with Genetic Algorithm (GA) to reduce the energy consumption from the network. Its operation is divided into rounds and each round consists of a setup phase and a steady-state phase. The BS determines the CHs and their locations with GA in the first phase, whereas the route from the source node to the BS is determined in the second phase. This approach allows a node to send data directly to the BS if the node’s distance to the BS is smaller than the distance to its CH. TDMA and CDMA schedules are used in this approach for intra-cluster and inter-cluster communication, respectively. Similarly, the approach in Ref. [36] also divides its operation into several rounds each of which again consists of a setup phase and a steady-state phase. In the setup phase, BS selects the CHs and forms clusters based on nodes’ location, residual energy, and the number of neighbors. The steady-state phase forwards data to the BS through an optimal route. Similar to the approach in Ref. [35], CHs in this approach also uses the TDMA schedule for intra-cluster communication. The proposal in Ref. [37] forms clusters of various sizes according to the residual energy and selects CHs with Shuffled Frog Leaping Algorithm (SFLA) [40]. Its operation is divided into two phases, namely, the cluster establishment and the data transmission phase. Selection of the CHs in the cluster establishment stage is an optimization problem. In the data transmission phase, it adopts a greedy approach to find the route from the source node to the BS. The operation of the approach presented in Ref. [38] can be divided into three phases, namely, the setup phase, neighbor finding phase, and the steady-state phase. In the first and second phase, nodes are classified into different layers and messages are broadcast to find the neighbors. This broadcast follows non-persistent Carrier Sense Multiple Access (CSMA) [41] protocol to access the medium. The third phase again can be divided into CHs selection, cluster formation, and data delivery. This approach uses fuzzy logic to select the CHs and ACO to find the optimal route for data delivery. Here, the input parameters are the number of neighboring nodes, residual energy, and the link quality. On the other hand, ACO uses distance to the BS, residual energy, delivery likelihood, and queue length to select the relay nodes. The approach presented in Ref. [39] proposes an unequal clustering and routing technique based on chemical reaction optimization [42]. It selects the CHs based on the optimization approach presented in Ref. [42] and assigns other nodes to the CHs based on a derived cost function. It also proposes a routing algorithm that is also based on the technique of Ref. [42]. The approach proposed in Ref. [16] combines an unequal clustering mechanism [43] to determine cluster sizes and a multi-objective immune algorithm [44] to produce a routing tree. The cluster sizes are determined based on the residual energy and distance to the BS. Thus, these approaches apply different heuristic optimization methods to find the CHs and to determine the cluster size. In these approaches, clusters are formed after the selection of CHs.

Among the recent works on hybrid unequal clustering approaches, the proposal in Ref. [15] focuses on equalizing the energy consumption from every cluster. For this purpose, it reverses the cluster formation steps by creating the clusters first then assigning the CHs to them. Hence, the three phases of clustering the network in this approach are performed in sequence- cluster formation, CH selection and data transmission. In the cluster formation phase, a Sierpinski triangle [15] is used to create smaller clusters near to the BS. While selecting the CHs, it considers node degree, residual energy, and distance to the BS. On the other hand, the proposal in Ref. [45] adopts a voting scheme to construct unequal clusters and selects the CHs based on the residual energy, topology, and transmission power. However, its CH selection is a distributed approach unlike the approach presented in Ref. [15].

The limitation of these approaches is that, their procedures of increasing the network lifetime prolong the declining state that introduces blind spot problem in the network. Declining state refers to the last stage of a network lifetime that begins when the nodes start dying. A long-lasting declining state in a given lifetime can degrade the performance of any clustering approach. The approach proposed in this paper tries to keep the declining state short by maintaining more equivalent residual energy in nodes after each round. For this purpose, it divides the monitoring area into several partitions before the selection of CHs that is similar to the approach presented in Ref. [15]. However, in the proposed approach, clusters are formed by cognitive partitioning instead of adopting fractals. In addition, path length connecting potential CHs and the BS is counted for the selection of CHs.

## 3. The Proposed Clustering Approach

This section describes the proposed clustering approach for reducing the blind spot problem in WSNs. The proposed approach is divided into the cluster formation phase and the CHs selection phase in order. The CHs selection phase is further divided into the candidate CH selection and the final CHs selection.

### 3.1. Network Model

A WSN can be represented by the graph *G* = (*V*, *E*), where *V* is the set of all sensors in the network and *E* = {(*u*, *v*)$\subset V|D_{u,v}\leq R\}$ represents the wireless connection between nodes. Here, $D_{u,v}$ is the distance between nodes *u* and *v* and *R* is the transmission range. It is assumed that:

- Homogeneous sensor nodes with the same functionality and capacity are deployed uniformly within a rectangle area. The BS is located at a distance from the monitoring area.
- Intra-cluster and inter-cluster communications are a single-hop and a multi-hop data transfer respectively that are conducted by the CHs, i.e., each CH must send the traffic to the next CH towards the BS.
- Only one CH is selected from each cluster in a round.
- As data aggregation is out of the scope of this paper, it is assumed that each event is captured by the nearest sensor only and each event generates an equal amount of data unit.

**Figure 1:**
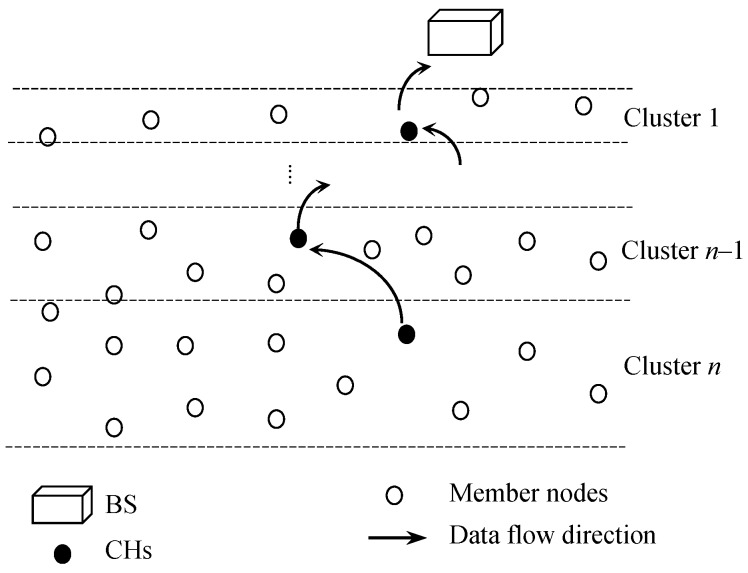
Architecture of a WSN with unequal clustering approach with serial partitioning.

**Figure 2:**
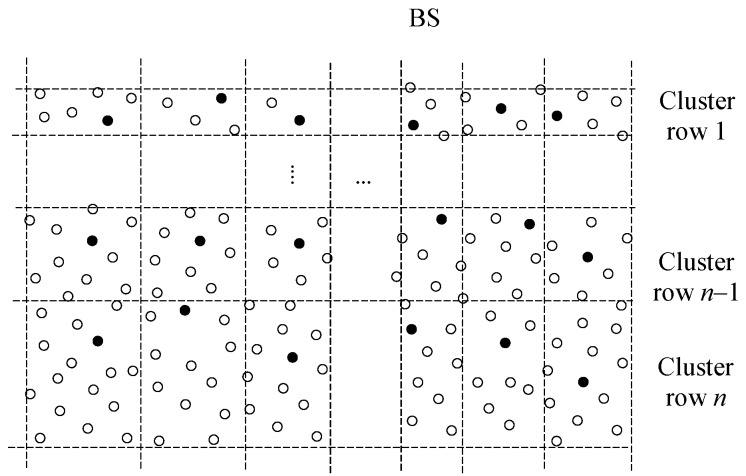
Potential unequal grid partitioning of a WSNs for future research direction.

Figure 1 illustrates the network with a BS, CHs, clusters, member nodes and data flow from CHs to the BS. It is assumed that the side of the monitoring area that is facing to the BS is equivalent to 2*R*. Otherwise the nodes had to use too many hops to reach their own CHs which results in energy-inefficiency inside the clusters. Considering the circular shape of wireless propagation model, cognitive grid partitioning of the monitoring area would be a perfect network model for facilitating monitoring rectangular area of any size which is illustrated in Figure 2. However, this research encourages the future works in grid partitioning and lays their groundwork with serial partitioning. Table 1 summarizes the important notations and their definitions used in the rest of the paper.

### 3.2. Details of the Proposed Approach

**Figure 3:**
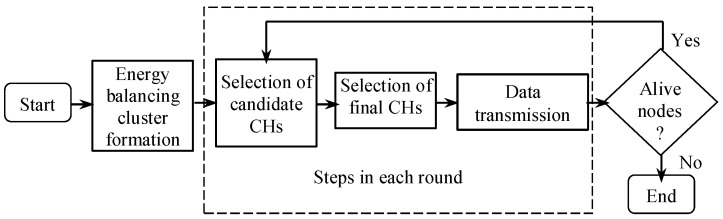
Stages of the proposed approach. After the cluster formation, BS enforces the operation of the network into rounds.

The proposed clustering approach can be divided broadly into (1) Energy-balancing cluster formation and (2) Repetitive operational rounds. After deployment, sensor nodes send their residual energy to the BS to facilitate the partition of the entire area *P* into *n* unequal sectors $p_{1}$, $p_{2}$, …, $p_{n}$ for balancing the energy during multi-hop data transmission. Here, $p_{1}$ is the closest sector to the BS and $p_{1}<p_{2}<\ldots <p_{n}$ in terms of size. After the partition, the operation of the entire network is divided into rounds. Each of these rounds again consists of three steps, namely, selection of candidate CHs, selection of final CHs from the candidates, and data transmission. Figure 3 illustrates the stages associated with the proposed approach which are described in the following subsections. However, data transmission is not covered as it is out of the scope of this research. Recent works have proved the effectiveness of cluster formation prior to the selection of CHs. Guiloufi et al. [15] have used a Sierpinski triangle for this purpose that ensures smaller cluster size near to the BS. However, fractal-based approaches fail to address any actual measurement of cluster size for balancing the energy consumption. The novelty of the proposed approach is that instead of forming clusters based on geometric fractals, it determines the actual size of $p_{i}$ in a cognitive way for balancing the energy consumption.

#### 3.2.1. Energy-Balancing Cluster Formation

As the first step towards the cluster formation, the BS divides the entire area equally into *n* serial partitions, namely $P_{1}$, $P_{2}$, …, $P_{n}$, where the separation line of any $P_{i}$ and $P_{i+1}$ is parallel to that edge of *P* that is closest to the BS. Now, assuming that each $P_{i}$ is a single node and *Z* is the total number of events occurred in *P* within a given time frame, the BS computes the energy loss at each $P_{i}$ for forwarding the corresponding data to $P_{j}$ using Equation (1). The probability of an event to occur in any partition $P_{i}$ can be defined as $P_{i}/P$. Thus, the total number of events occurred in that area becomes *Z*($P_{i}/P$). Assuming one event generates one data unit, any partition $P_{i}$ must receive all the data from $P_{i+1}$ and transfer to $P_{i-1}$ after accumulating its own data. Hence, $P_{i}$ must receive and transfer more data than $P_{i+1}$, i.e., energy loss in $P_{i}$ is greater than that in $P_{i+1}$. Therefore, the relation between energy losses in each partition becomes: $e_{n}<e_{n-1}<\ldots <e_{1}$. Equation (2) shows the energy loss of $P_{n}$ which, being the farthest partition, is not burdened with receiving any data from other partitions. The BS now determines the percentage of the area to adjust from $P_{i}$ using Equations (3) and (4).

(1) $$e_{i}=Z\left(\frac{\sum_{l=i-1}^{n}P_{l}}{P}\right)\cdot C_{r}+Z\left(\frac{\sum_{l=i}^{n}P_{l}}{P}\right)\cdot D_{i,j}^{\lambda }C_{s}$$

(2) $$e_{n}=Z\left(\frac{P_{n}}{P}\right)\cdot D_{n,n-1}^{\lambda }C_{s}$$

(3) $$d_{i}=\left(\frac{1}{n}-\frac{e_{i}}{\sum e}\right)\times 100$$

(4) $$p_{i}=P_{i}+P_{i}\times d_{i}$$

Here, $d_{i}$ denotes the deviation of energy in $P_{i}$ from the equidistributed energy in percentage. The proposed approach tries to minimize this deviation by adjusting the area of $P_{i}$ by $d_{i}$% in Equation (4). The total energy loss of $P_{i}$ within a given time frame can be factorized into three components such as the energy loss for receiving data from $P_{i+1}$, sending the same amount of data to $P_{i-1}$ and sending the data of $P_{i}$’s local events to $P_{i-1}$. As $P_{i}$ must receive the data from $P_{i+1}$ and transfer to $P_{i-1}$, the BS focuses on controlling $P_{i}$’s local events to reduce its energy deviation. The more the number of local events in $P_{i}$, the more its energy loss. Again, the number of local events is proportional to the area because of the uniform distribution of nodes. Hence, the adjusted area $p_{i}$ is expected to have no or small deviation in energy from the equidistributed energy in percentage.

#### 3.2.2. Selection of the Candidate CHs and Final CHs

The BS selects CHs after the formation of *n* clusters in the network. For this, the BS selects a set of candidate nodes $S_{i}$ comprising all $c_{i_{j}}$ in $p_{i}$ such that *w*($c_{i_{j}}$) <T, where *T* = ($Min_{w_{i}}$ + $Min_{w_{i}}\times m$). Here, $Min_{w_{i}}$ is the minimum weight in $p_{i}$ and *m* is a predefined value for determining *T*. The value of *m* determines the tolerance of the proposed approach to select the candidate CHs. If m=0, only the nodes with minimum weight are selected as candidates. In this case, there is a high probability that only one node is selected as the candidate CH from a cluster which eventually becomes the final CH. Here, the purpose of *m* is to select several equivalent weighted nodes as candidate CHs. if m=1, nodes with weight higher than the two times of the minimum weigh get selected. Point to be noted that residual energy can be an important factor to calculate the weight. The lower the residual energy, the higher the weight is. Hence, if all other factors remain constant, m=1 will include those nodes into the candidate list also who have residual energy as half as the highest weighted ones. Eventually, one of these low energy nodes may get selected as the final CH in the cluster which may cause an imbalance in the residual energy in the cluster. Hence, it is always a good practice to keep the value of *m* (0<m<1) towards 0. One of the advantages of *m* is that it keeps the number of candidate CHs unguaranteed. Consequently, if a node has weight way below than the average in its cluster, this approach can force the node to serve as candidate CH and eventually the final CH for several rounds until the node’s energy get reduced and the weight comes to the average value. The weight function *w* is defined in Equation (5) for any node *i*.

(5) $$w\left(i\right)=a_{1}F_{i_{1}}^{\alpha }+a_{2}F_{i_{2}}^{\beta }+\ldots +a_{q}F_{i_{q}}^{\gamma }$$

**Figure 4:**
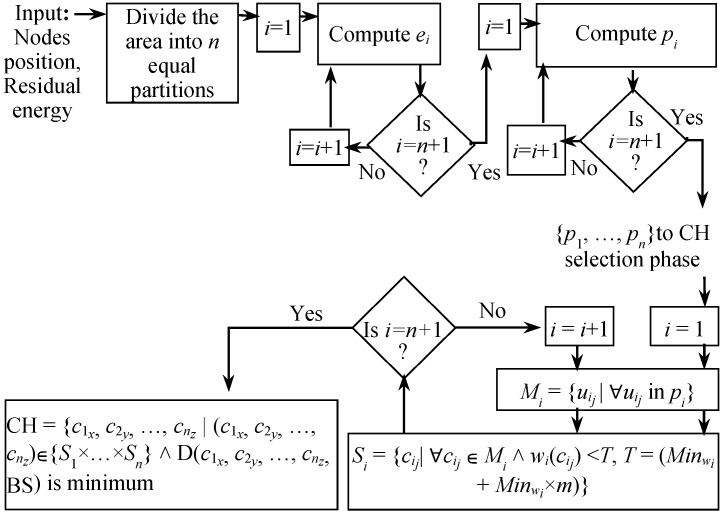
Proposed cognitive partitioning and single round CH selection approach.

Here, $a_{1}$, $a_{2}$, …, $a_{q}$ are coefficients and $F_{i_{1}}$, $F_{i_{2}}$, …, $F_{i_{q}}$ are the associated factors with *i*, e.g., residual energy, number of replaceable nodes [46], nodes degree, etc., and α, β, …, γ are the orders of $F_{i_{1}}$, $F_{i_{2}}$, …, $F_{i_{q}}$. From $\{S_{1}$, $S_{2}$, …, $S_{n}\}$, the BS selects $\{c_{1_{x}}$, $c_{2_{y}}$, …, $c_{n_{z}}\}$ as CHs for $p_{1}$, $p_{2}$, …, $p_{n}$ such that $D_{c_{1_{x}},c_{2_{y}},\ldots ,c_{n_{z}},BS}$ is minimum. Hence, according to the rule of product, the BS checks $\prod_{i=1}^{n}|S_{i}|$ values to find the CHs that yield the least distance. The steps associated with the proposed clustering approach is illustrated in Figure 4 and are summarized as:

1. Serially divide the monitoring area into equal partitions.
2. Adjust the monitoring area based on the expected energy consumption and yield $p_{1}$, $p_{2}$, …, $p_{n}$.
3. For each monitoring area $p_{i}\in \{p_{1}$, $p_{2}$, …, $p_{n}\}$ repeat steps 4 and 5.
4. Create set $M_{i}$ comprising all nodes in $p_{i}$.
5. Create candidate set $S_{i}$ comprising only that elements of $M_{i}$ for which calculated weight is less than *T*, where *T* = ($Min_{w_{i}}$ + $Min_{w_{i}}\times m$).
6. Find the combination of candidate CHs that yields minimum cumulative distance with the BS by taking exactly one node from each $S_{i}$.

**Figure 5:**
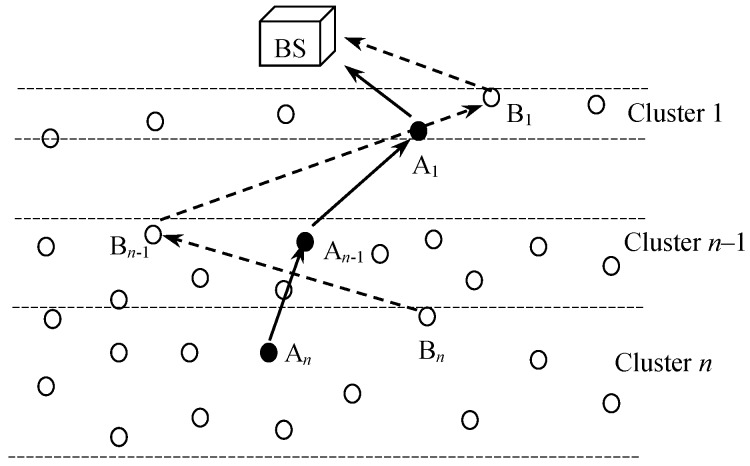
Significance of total path connecting all CHs instead of individual paths from CHs to the BS.

A majority of the previous related works have considered individual distances to the BS while selecting the CHs, whereas the proposed approach counts the total path to BS connecting all potential CHs. The significance of considering the total path can be understood with Figure 5. If all other factors except the distance to the BS were kept constant, the majority of existing clustering approaches would select $\{B_{n}$, $B_{n-1}$, …, $B_{1}\}$ as CHs because of their short individual distances to the BS. This may lead the network losing more power in a multi-hop communication due to a long cumulative distance to the BS through all CHs. The proposed approach eradicates the problem by selecting those candidate nodes as the CHs ($\{A_{n}$, $A_{n-1}$, …, $A_{1}\}$ in Figure 5) that yield the lowest cumulative distance to the BS.

### 3.3. Directions for Heterogeneous WSNs

Although this paper considers homogeneous WSNs for its purpose, heterogeneous WSNs can also adopt the proposed approach with little modification. In homogeneous WSNs, all nodes are considered to be the same in terms of residual energy, radio transmission capability, processing power, etc. However, heterogeneous WSNs consists of nodes with different residual energy, processing, and transmission power. As this proposal focuses on balancing residual energy strictly, its candidate CHs selection procedure (Equation (5)) is designed to choose only those nodes which are superior than others in terms of considered factors. In homogeneous WSNs, all nodes have the same capability of becoming a candidate CH at the beginning. Nodes served as CHs and left with less energy have low chance of being a candidate CHs in the subsequent rounds until the residual energy of other nodes is reduced to the equivalent level. Similarly, in heterogeneous WSNs, all advanced nodes are forced to be selected as candidate CHs until their residual energy is reduced to the average. Again, candidates having values within a calculated range is considered only that guarantees the most resourceful nodes compete to be the final CHs. For example, in a given cluster $p_{i}$, if the two lowest weights are calculated as Δ and δ and δ<(δ+δ×m)<Δ, node with weight value Δ will not get a chance to be a CH until the other node loses energy by serving as a CH and their new weights become $\Delta^{\prime }$ and $\delta^{\prime }$ where $\delta^{\prime }<\Delta^{\prime }<\left(\delta^{\prime }+\delta^{\prime }\times m\right)$.

Hence, for adopting the proposed approach in heterogeneous WSNs, besides residual energy, replaceable nodes, and nodes degree other factors e.g., processing power, transmission power, etc., should be considered also. Furthermore, it is recommended that nodes of all types are uniformly distributed such that each cluster gets equivalent ratio of normal and advanced nodes.

## 4. Evaluation of the Proposed Scheme

### 4.1. Simulation Parameters and Energy Consumption Model

The proposed clustering approach has been simulated with MATLAB. The energy consumption model (Equation (6)) of the transceiver unit is the same as the approach given in Ref. [15].

(6) $$E_{c}\left(i\right)=E_{R_{x}}\left(k\right)+E_{T_{x}}\left(k,D\right)$$

$$E_{R_{x}}\left(k\right)=E_{elec}\times k$$

$$E_{T_{x}}\left(k,D\right)=e_{amp}\times k\times D^{\lambda }$$

(7) $$w\left(i\right)=\frac{E_{c}\left(i\right)}{E_{n}}$$

Here, $E_{elec}$ is the energy dissipation to run the receiver circuitry for *k* bits of data and $e_{amp}$ is the energy consumption by transmitter power amplifier to send the same bits of data over a distance *D*. The weight of any node *i* is determined using Equation (7). Here, $E_{c}\left(i\right)$ and $E_{n}$ refer to the consumed energy and the initial energy of the node *i*. The list of simulation parameters and the cluster properties generated by the proposed approach are shown in Table 2 and Table 3, respectively.

### 4.2. Network Lifetime with the Proposed Approach

**Figure 6:**
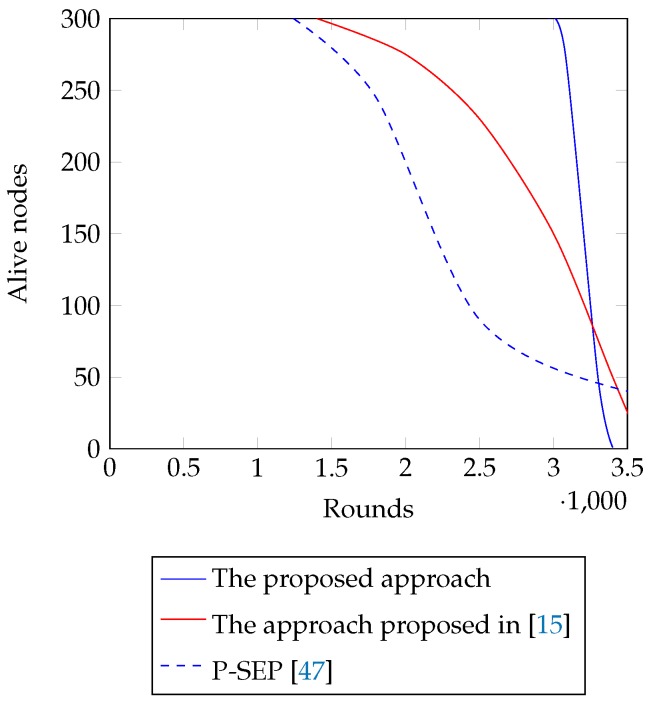
Comparison among the proposed approach, the approach presented in Ref. [15], and P-SEP [47] in terms of number of alive nodes per round.

Figure 6 shows the number of alive nodes per round with the proposed approach and with the approaches presented in Refs. [15,47]. From the figure, it is seen that nodes start dying at about Round 1400 in Ref. [15] and 1200 in Ref. [47]. At Round 2000, the network loses about 8% and 16% of their sensor nodes with Refs. [15,47] respectively. Again, 50% of the sensor nodes fall into the dead state with Ref. [15] at Round 3250 that raises to 90% at Round 3500. However, with the approach proposed in Ref. [47], the same incidents occur at Round 2200 and 3700 respectively. The proposed approach, on the other hand, shows a steeper curve in terms of alive nodes per round. However, nodes in the proposed approach survive more rounds than the proposal in Refs. [15,47]. The proposed approach keeps all nodes alive until the Round 2971. Unlike the approach of Refs. [15,47], it loses 8% of the nodes at Round 3080 and 50% at Round 3250. Soon after the death of 50% of the nodes, the network survives only for a few more rounds that end with the last node death at Round 3400. Table 4 represents a comparison among the proposed approach and the approach presented in Refs. [15,47] in terms of First Node Dead (FND), Half Node Dead (HND), and Last Node Dead (LND). The proposed approach is more efficient in terms of FND and HND. Although it falls behind the approach presented in Refs. [15,47] for LND, it successfully minimizes blind spot problem by minimizing the duration between FND and LND.

### 4.3. Reduction of Blind Spot Problem

At the beginning of the operation, the network shows good performance by capturing the desired number of events per unit of time. However, towards the end of the lifetime, the network experiences death of its constituent nodes which results in inability to capture events in certain places. This scenario is described as blind spot problem which results in degradation of network performance in terms of number of captured events per unit of time. The sole reason of this problem lies on the fact that a few nodes die out quickly in the network due to unbalanced energy consumption. The more unbalanced the energy consumption is, the more quickly nodes start dying in the network. On the other hand, majority of the recent clustering approaches focus on enlarging the network lifetime without incorporating a robust energy-balancing technique. This results in a long duration between FND and LND, i.e., the network suffers from event capturing inability or blind spot problem for a long time. To reduce this sufferance, this paper focuses on adopting a mechanism that forces a balanced energy consumption in each round for both nodes and clusters.

Figure 6 also shows the duration of blind spot problem in the network with the proposed approach and with the approaches presented in Refs. [15,47]. From the figure, it is seen that the round count for FND is 1400 with [15] and 1200 with [47]. For LND, it is Round 3600 with [15] and with [47], about 10% nodes survived till the end of the simulation as they were considered advanced and more resourceful than other nodes. Since all normal nodes died at about Round 3700, the networks suffered from blind spot problem for 2500 rounds with [47]. Similarly, with [15], the network suffered from the same problem for 2200 rounds according to the simulation. On the contrary, the network experienced FND and LND at Round 2971 and 3400 respectively that allowed the blind spot problem to persist for 429 rounds only. Thus, the proposed approach reduces the blind spot problem in the network.

### 4.4. Balanced Energy Consumption

**Figure 7:**
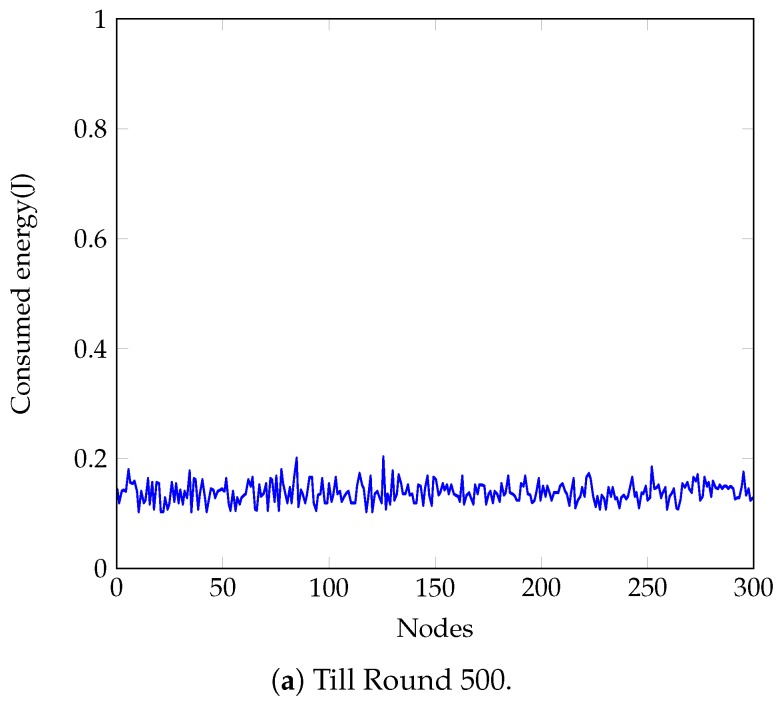

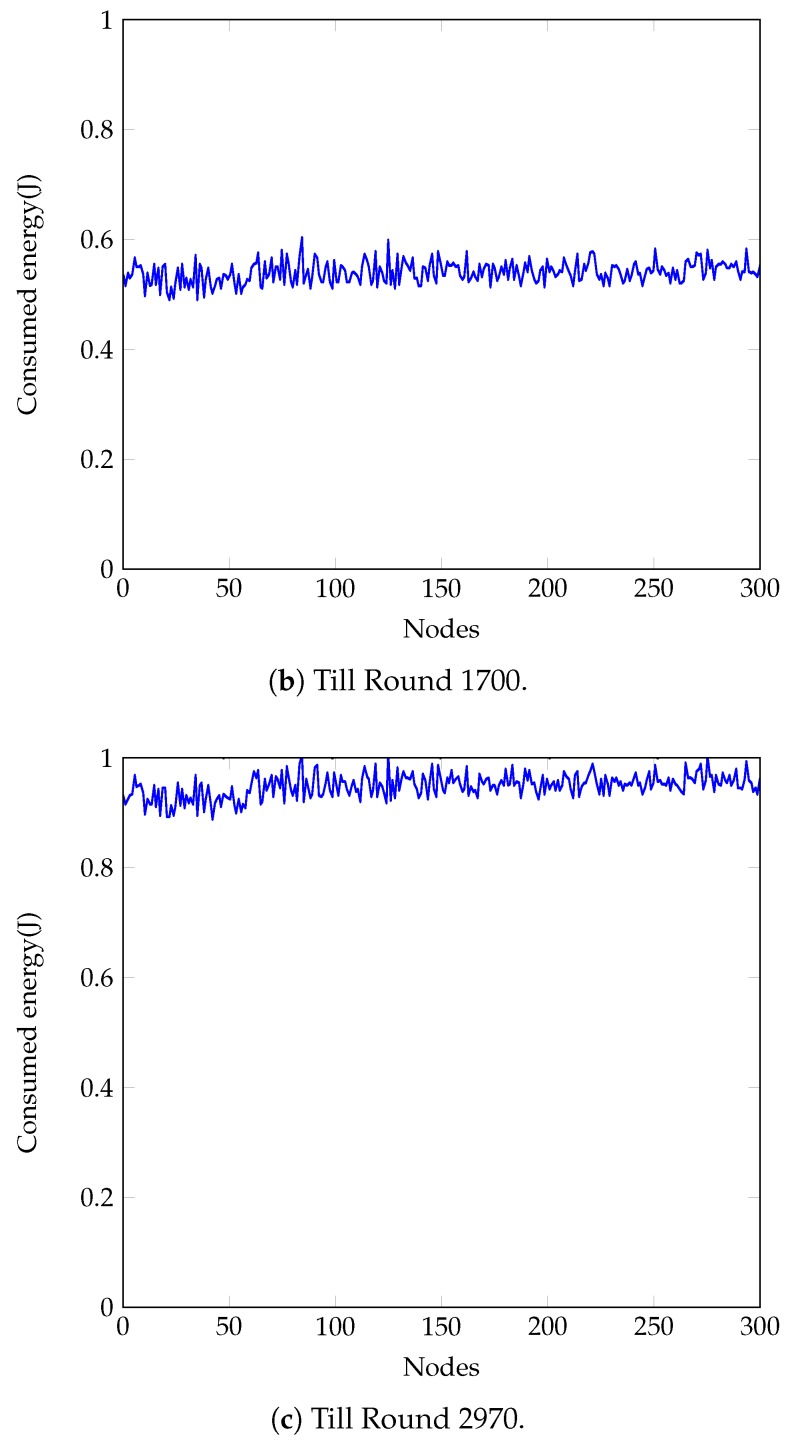
Energy consumption till different Rounds of the proposed approach.

**Figure 8:**
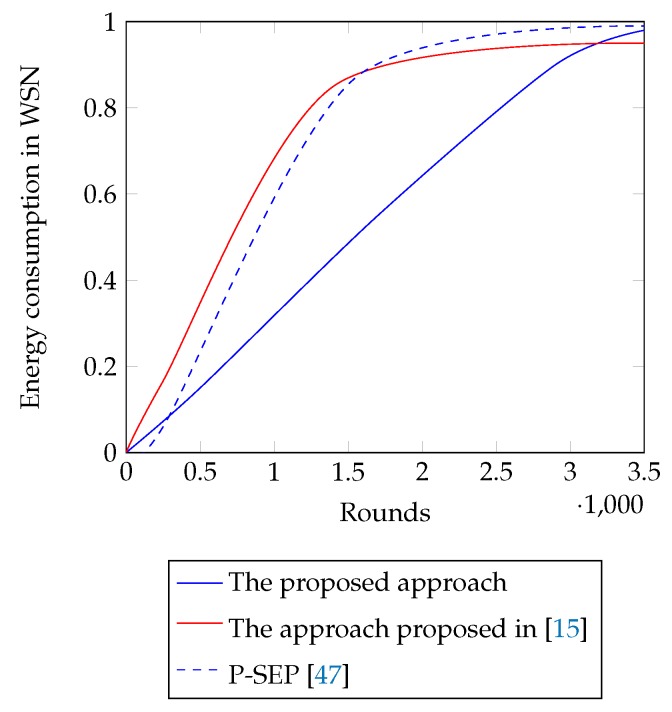
Average energy consumption of the WSN in each round.

**Figure 9:**
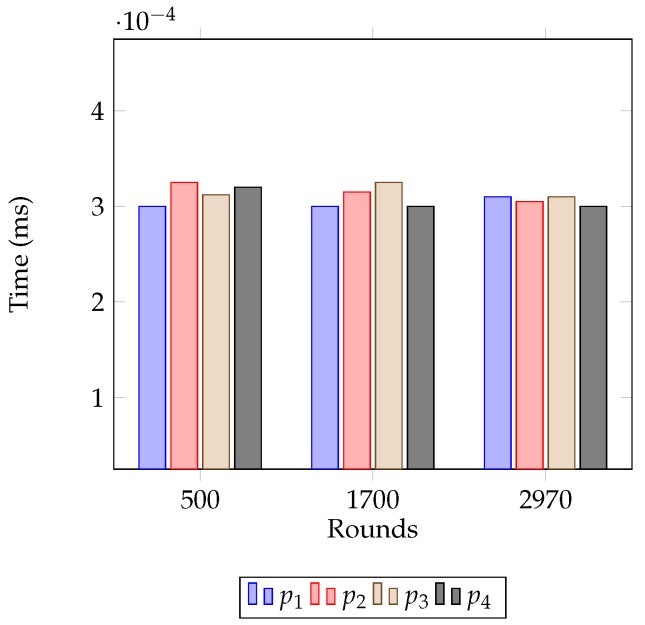
Average energy consumption in each cluster with the proposed approach at Rounds 500, 1700 and 2970.

Figure 7 represents the consumed energy of each node till Rounds 500, 1700 and 2970 with the proposed approach. From the figure, it is seen that the values are equivalent for all nodes in any particular round and they fluctuate ±0.05 J from the average value of that round. At Round 1700, the values remain within the range of 0.5 J and 0.6 J, i.e., about 55% of the initial energy is consumed until this round. At Round 2970, the values fluctuate between 0.89 J to 1 J; hence, from this round, nodes start falling into the dead state. Table 5 summarizes the energy consumption of the approach proposed in Refs. [15,47] till Rounds 250, 700, and 1500. These approaches consume 50% of the nodes’ initial energy at about Round 700. The energy consumption in Ref. [15] varies ±0.07 J from the average value in early rounds and raises up to ±0.13 J in later rounds. It justifies that the proposed approach is about 50% more efficient than [15] in terms of balancing the residual energy after the completion of a round. Again, the energy consumption in Ref. [47] varies ±0.15 J from the average value in early rounds and raises up to ±0.7 J in later rounds which makes the proposed approach way more efficient. Average energy consumption of the network with the proposed approach and with the approached presented in Refs. [15,47] is shown in Figure 8. From the figure it is shown that nodes experience sustained energy for more rounds than aforementioned approaches. Figure 9, on the other hand, shows average energy consumption in each cluster at Rounds 500, 1700 and 2970 with the proposed approach. From the figure, it is seen that the average energy consumption in any cluster varies between 3 × 10${}^{-4}$ J and 3.5 × 10${}^{-4}$ J. Hence, this figure also indicates that the proposed approach maintains an equivalent residual energy in all clusters throughout the lifetime of the network.

### 4.5. Distribution of Dead and Alive Nodes

**Figure 10:**
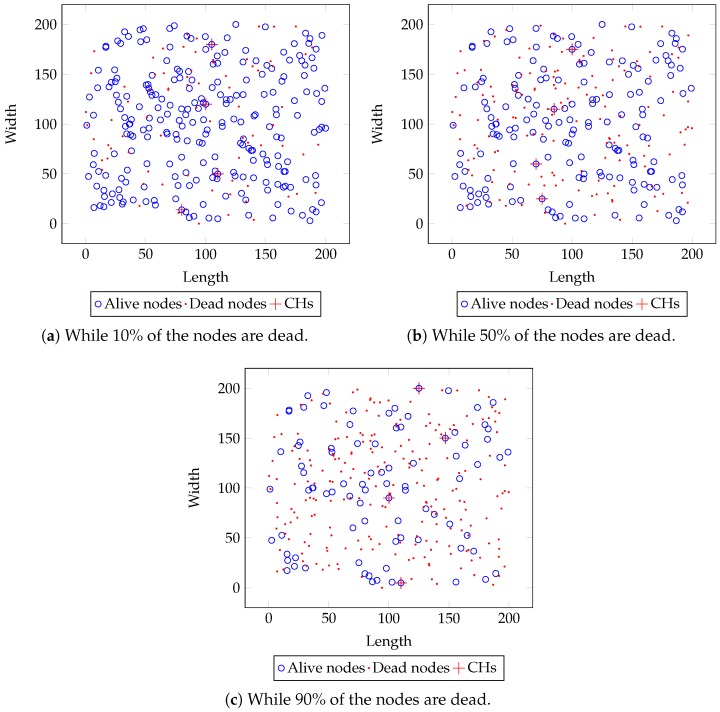
The distribution of dead and alive nodes in the network with the proposed approach.

Distribution of dead and alive nodes is also an important aspect to consider while evaluating the performance of a clustering approach. Beside of reducing the duration of the declining state, the clustering approach should ensure the uniform occurrence of node’s death throughout the network during this state. This maintains the consistency between the alive nodes and the probability of capturing an event during the declining state. Figure 10 shows dead and alive nodes distribution during the declining state when 10%, 50%, and 90% nodes are dead in the network. From the figure, it is inferred that the proposed approach maintains a uniform distribution of alive nodes during the declining state.

### 4.6. Discussion

In terms of alive nodes per round (Figure 6), the proposed approach shows a steeper curve than the approaches proposed in Refs. [15,47]. However, with the proposed approach, FND occurred at the later rounds than the approaches in Refs. [15,47]. This is because the proposed approach guarantees the shortest path connecting each CH to the BS. Thus, nodes lose less energy while serving as CHs. Again, the cognitive partitioning of the monitoring area enables each cluster to maintain a member size that ensures equivalent energy consumptions in them. Also, from the Table 4, it is seen that the average efficiency of the proposed approach is more than the proposal in Ref. [15] in this regard.

From Table 5, Figure 7 and Figure 8, it is seen that the proposed approach is more efficient in terms of stabilizing nodes residual energy. Furthermore, Figure 9 shows small differences in average energy consumption in each cluster at different rounds which indicates a balanced energy consumption among the clusters also. Hence, nodes are left with small and equivalent energy while they enter the declining state. With the small remaining energy network hardly runs for a few rounds, i.e., the duration of the declining state becomes small that reduces the blind spot problem in the network.

From Figure 10, it is again seen that the proposed approach maintained a uniform distribution of alive nodes during the declining state. Without a uniform distribution of dead and alive nodes, the blind spot problem would be acute in declining state. Hence, the distribution of dead and alive nodes becomes a significant factor while designing blind spot reducing approaches. The proposed approach not only reduces the duration of the declining state but also ensures the uniform occurrence of node’s death which maintains the probability of capturing events with respect to the alive nodes throughout the lifetime.

## 5. Conclusions

In this paper, we have proposed a new unequal clustering approach for WSNs that minimizes the blind spot problem while prolonging the network lifetime. This approach promises equivalent and least energy consumption from the clusters in each round. For this purpose, the clusters are formed by dividing the monitoring area into multiple partitions in a cognitive way. Such a partitioning approach ensures the consumption of energy to be equivalent in each cluster. Furthermore, the proposed approach adopts a two-layered scrutinization while selecting the CHs. This ensures the least energy consumption from the network and prolongs the steady state. In addition, nodes are left with small energy before entering the declining state as they maintain equivalent energy consumption throughout the steady state. Consequently, the declining state gets a short duration that reduces the blind spot problem in WSNs.

Future work for extending the proposed approach would entail the cognitive partitioning of the network to support scalability. The authors have already developed a Blockchain-based protocol suite for WSNs [48] that adopts the proposed clustering approach of this paper. Besides, considering different node matrices for the selection of candidate CHs, finding an efficient optimization algorithm for the selection of final CHs, and cognitive grid partition of WSNs would also be some good future research issues.

## Figures and Tables

**Table 1:** Notations and their definitions.

Notations	Definitions
*N*	Number of nodes
*n*	Number of clusters, i.e., number of CHs
*P*	A rectangular monitoring area
$P_{i}$	*i*th equal partition of the monitoring area
$p_{i}$	Adjusted area of $P_{i}$ for energy balancing
*Z*	Number of events in *P* within a time frame
$C_{r}$ and $C_{s}$	Units of energy consumed for running receiver and sender circuitry for one data unit
$D_{i,j}$	Distance between node *i* and node *j*
$D_{i,k_{1},k_{2},\ldots ,k_{k},j}$	Distance between node *i* and node *j* via $k_{1}$, $k_{2}$, …, $k_{k}$
λ	Path loss exponent
$S_{i}$	Set of candidate CHs from $p_{i}$
$c_{i_{j}}$	*j*th candidate nodes in $p_{i}$ ($\in S_{i}$)
*w*($c_{i_{j}}$) and *w*($p_{i}$)	Weight of node $c_{i_{j}}$ and summation of all node’s weight in $p_{i}$

**Table 2:** Simulation parameters.

Parameters	Values
*N*	300
*P*	200 m × 200 m
*m*	0.02
$E_{elec}$	50 × $10^{-9}$
$e_{amp}$	10 × $10^{-12}$
*k*	4000
$E_{n}$	1 J
BS coordinate	(100, −50)
*n*	4
λ	2

**Table 3:** Size of the clusters.

Clusters	Length (m)	Width (m)	Intermediate Clusters to the BS in Order	Number of Member Nodes
$p_{1}$	200	41.07	-	62
$p_{2}$		46.78	$p_{1}$	71
$p_{3}$		52.05	$p_{2}$, $p_{1}$	79
$p_{4}$		59.65	$p_{3}$, $p_{2}$, $p_{1}$	88

**Table 4:** The proposed approach vs. the approach presented in Ref. [15] vs. P-SEP [47] in terms of FND, HND, and LND.

Clustering Approach	FND	HND	LND
The proposed approach	2971	3250	3400
The approach presented in Ref. [15]	1400	2800	3600
P-SEP [47]	1242	2163	NND *

* Node never dies.

**Table 5:** Nodes energy consumption with the approach proposed in Ref. [15].

Rounds	Min. (J)			Max. (J)		
	Ref. [15]	Ref. [47]	Proposed	Ref. [15]	Ref. [47]	Proposed
250	0.01	0.05	0.01	0.15	0.27	0.11
700	0.35	0.30	0.17	0.52	0.61	0.28
1550	0.74	0.67	0.42	1.00	1.00	0.50
